# Proceedings from the 2023 transdisciplinary conference for future leaders in precision public health “Applying Implementation Science to Precision Public Health”

**DOI:** 10.1186/s12919-025-00332-6

**Published:** 2025-07-03

**Authors:** Katherine W. Saylor, Caitlin G. Allen, Jarrod Marable, Cason Whitcomb, Dana Lee Olstad, Julia Steinberg, Amelia Smit, Erin Turbitt, Kimberly Foss, Latrice Landry, Megan C. Roberts

**Affiliations:** 1 University of Pennsylvania, Philadelphia, PA USA; 2 Medical University of South Carolina, Charleston, SC USA; 3 University of North Carolina, Chapel Hill, NC USA; 4 University of Calgary, Calgary, AB, CA Canada; 5 The Daffodil Centre, The University of Sydney, a joint venture with Cancer Council NSW, Sydney, NSW Australia; 6 University of Sydney, Camperdown, NSW, AU Australia; 7 University of Technology Sydney, Ultimo, NSW, AU Australia

## Abstract

Precision public health (PPH) approaches use big data to inform tailored, population-level interventions. The field has roots in genomics, but it has expanded to encompass data-informed public health programs across various types of data or applications. The Precision Public Health Network hosted a 2023 conference focused on implementation science—the study of how to integrate PPH programs into practice. Some implementation needs that emerged across speakers included establishing robust evidence of clinical utility and feasibility, disseminating clinical best practices through guidelines and tools for providers, sharing tools or information to reduce duplicated efforts across settings, and considering context-specific factors. Considering feasibility, setting-specific factors, and meaningful engagement with relevant user groups throughout the research and implementation process are critical to the successful and sustainable implementation of PPH programs. The Network also hosted an interactive workshop to generate ideas and ongoing collaboration on essential outcomes or data measures for PPH programs, and strategies to center health equity in PPH. This conference and workshop are part of the ongoing work of the PPHN to convene experts across disciplines and settings, share knowledge, and galvanize the field of PPH.

## Introduction

Precision public health (PPH) has been defined as the integration of big data and population-based strategies to provide “the right intervention to the right population at the right time.” [1] There is growing interest in precision approaches to improving the impact of public health investments across a range of public health applications. Research efforts have shown promise in the development of interventions that stratify populations by risk and tailor interventions to specific subpopulations. As PPH interventions are being developed, it is crucial to think strategically about how to move from research studies and pilot programs to sustainable and equitable implementation.

To address this need, an interdisciplinary group of PPH researchers convened a virtual conference and workshop, “*Applying Implementation Science to Precision Public Health.*” The conference served to share lessons learned, and the workshop furthered collaborations on measuring the impact of PPH programs and centering equity in the design and implementation of such programs.

This conference and workshop were organized by the Precision Public Health Network (PPHN), an international interdisciplinary group of researchers invested in studying and promoting PPH. The PPHN was launched in 2021, with the first virtual conference “*Transdisciplinary Conference for Future Leaders in Precision Public Health.”* The first conference convened early-to-mid-career researchers, established a set of shared research priorities, and launched collaborative projects. Today, PPHN members represent diverse disciplines (including health services research, implementation science, bioethics, public policy, genomics, epidemiology, and nutrition science). Members range from graduate students to early- and mid-career faculty, and are located across the U.S., Canada and Australia. The PPHN provides opportunities for knowledge sharing through events open to the public, training workshops, and ongoing member-driven research projects.

In 2021, PPHN members identified three priority areas that have shaped the network’s activities: implementation science, health equity, and capacity building. Each theme was to be the focus of a future conference, starting with the 2023 virtual workshop on implementation, a 2024 conference on health equity hosted by the Medical University of South Carolina, and a 2025 conference on capacity building hosted by the University of Technology Sydney. These themes also guide the collaborative projects of PPHN members.

The 2023 conference focused on implementation science—how to deliver interventions and ensure that these interventions reach target populations and improve public health. Implementation science is the study of methods to promote the adoption and integration of evidence-based practices into routine health care and public health settings to improve health impact [2]. The goal of the conference was to share information on recent implementation science studies, and to discuss implementation barriers, facilitators, and best practices with potential for broader application. A broader goal of the conference was to expand the reach of the PPHN by encouraging networking and collaboration.

The virtual workshop, held a week after the 2023 conference, had two primary goals: to gather input on an ongoing PPHN project on how to measure the impact of PPH interventions, and to generate strategies for engaging underserved populations in PPH research and practice. The workshop was structured to generate ideas through active participation and to set up opportunities for participants to join ongoing projects.

The conference agenda and recorded presentations are available at https://pharmacy.unc.edu/pphnconference/.

## Methods

### Conference planning, communication, and day-of logistics

The virtual day-long conference and half-day workshop were organized by a planning committee and a dedicated working group focused on the workshop. The international planning committee met virtually and iteratively monthly or as needed. They developed the program and speaker list based on members’ knowledge and consultation with other PPHN members or informal conversations with colleagues. Committee decisions were made by consensus. Abstracts were solicited for the virtual poster session and were reviewed and scored by planning committee members for inclusion and award consideration, based on relevance to the conference theme, quality, and scientific contribution. Advertising was conducted via network listservs, social media, and direct outreach, with weekly posts and speaker highlights shared leading up to the event.

The events were hosted on Zoom. Chat features were enabled, and attendees could unmute to engage during Q&A sessions. During the conference, registered attendees received reminder emails 15 min before each session. The three-hour workshop took place one week after the virtual conference. Workshop facilitators led the sessions using the Zoom functionalities of Chat and Breakout Rooms to engage the virtual participants.

### Conference and workshop registrants

A total of 129 people registered for the virtual conference and 83 people registered for the workshop. Registrants self-reported their public health discipline, which included: health services research (*n* = 53, 41.09%), health behavior (*n* = 25, 19.38%), epidemiology (*n* = 29, 22.48%), health policy (*n* = 14, 10.85%), biostatistics (*n* = 4, 3.10%), and environmental health (*n* = 2, 1.55%). Most registrants were from North America (*n* = 96, 74.42%) followed by Australia (*n* = 10, 7.75%) and Europe (*n* = 10, 7.75%) (Table 1).

**Table 1:** Demographics of participants

	**All registrants (*n*** **=129)**	**Conference (*n*** **=127)**	**Workshop (*n*** **=98)**
**Gender**	**n (%)**		
**Female**	84 (65.12%)	82 (64.57%)	64 (65.31%)
**Male**	23 (17.83%)	23 (18.11%)	16 (16.33%)
**Unknown**	1 (0.78%)	1 (0.79%)	0 (0.00%)
**Prefer not to identify**	21 (16.28%)	21 (16.54%)	18 (18.37%)
**Race**	**n (%)**		
**Asian**	24 (18.60%)	23 (18.11%)	17 (17.35%)
**Black or African American**	12 (9.30%)	12 (9.45%)	11 (11.22%)
**White or Caucasian**	59 (45.74%)	58 (45.67%)	39 (39.80%)
**Multiple Races**	5 (3.88%)	5 (3.94%)	5 (5.10%)
**Prefer not to identify**	29 (22.48%)	29 (22.83%)	26 (26.53%)
**Geography**	**n (%)**		
**Africa**	6 (4.65%)	6 (4.72%)	5 (5.10%)
**Asia**	5 (3.88%)	5 (3.94%)	5 (5.10%)
**Australia**	10 (7.75%)	9 (7.09%)	10 (10.20%)
**Europe**	10 (7.75%)	10 (7.87%)	8 (8.16%)
**North America**	96 (74.42%)	95 (74.80%)	68 (69.39%)
**South America**	2 (1.55%)	2 (1.57%)	2 (2.04%)
**Career Status**	**n (%)**		
**Early-Career (<10 years post-degree attainment)**	44 (34.11%)	43 (33.86%)	36 (36.73%)
**Mid-Career (>10 - <25 years post-degree attainment)**	35 (27.13%)	35 (27.56%)	31 (31.36%)
**Late-Career (>25 years post-degree attainment)**	14 (10.85%)	13 (10.24%)	10 (10.20%)
**Prefer not to identify**	3 (2.33%)	3 (2.36%)	1 (1.02%)
**Student/Trainee**	33 (25.58%)	33 (25.98%)	20 (20.41%)
**Public Health Discipline**	**n (%)**		
**Biostatistics**	4 (3.10%)	4 (3.15%)	3 (3.01%)
**Environmental Health**	2 (1.55%)	2 (1.57%)	2 (2.04%)
**Epidemiology**	29 (22.48%)	28 (22.05%)	24 (24.49%)
**Health Behavior**	25 (19.38%)	25 (19.69%)	16 (16.33%)
**Health Policy**	14 (10.85%)	14 (11.02%)	11 (11.22%)
**Health Services Research**	53 (41.09%)	52 (40.94%)	41 (41.84%)
**Not Reported**	2 (1.55%)	2 (1.57%)	1 (1.02%)

### Post-conference activities

After the conference and workshop, evaluation surveys were distributed via email to all attendees following the close of conference activities. The evaluation included overall assessments and specific questions for each session. Virtual thank you notes were sent to all speakers, and a follow-up planning committee meeting was scheduled one month after the event to review evaluation data, and to plan future conferences, training programs, and other collaborations. Attendees received two reminder emails to complete the evaluation survey before being considered lost to follow-up. All registered registrants were included on a network listserv to be notified about future PPHN events.

## Results

### Virtual conference overview

The conference featured prominent speakers, beginning with a welcome from Dr. Megan Roberts, an opening presentation by Dr. Muin Khoury, Director of the Centers for Disease Control and Prevention (CDC) Office of Genomics and Precision Public Health, and a plenary session by Dr. Bruce Korf, Associate Dean for Genomic Medicine and Distinguished Professor of Genetics at the University of Alabama at Birmingham. Networking and expert-led sessions were held throughout the day with presentations from Dr. Nora Pashayan, Professor of the Epidemiology of Aging, University of Cambridge; Dr. John Mathers, Professor of Human Nutrition, Newcastle University; Dr. Michael Kosorok, Distinguished Professor, Department of Statistics and Operations Research, University of North Carolina at Chapel Hill; Dr. Sarah Knerr, Assistant Professor of Health Systems and Population Health, University of Washington; Dr. Deborah Cragun, Associate Professor and Director of Genetic Counseling, University of South Florida; and Dr. Stephanie Best, Associate Professor of Implementation Science, University of Melbourne. The day concluded with a closing session by Dr. Rachel Shelton, Professor of Sociomedical Sciences, Columbia University, and a summary by Dr. Erin Turbitt, Senior Lecturer in Genetic Counseling, University of Technology, Sydney.

#### Opening and plenary 1

##### Dr. Muin Khoury, “Opening, Definitions, and Grounding”

Dr. Khoury set the stage for the conference with an overview of the fields of public health genomics and PPH. He noted that 2023 marks the 25^th^ anniversary of the founding of the CDC Office of Genomics and Precision Public Health (formerly called the Office of Public Health Genomics) with the mission of enabling public health initiatives to integrate genomics into actions that prevent disease, reduce health disparities, and improve health. Dr. Khoury reflected on the evolution of the term PPH, and how the PPH’s emergence demonstrates a transition from the more narrow focus of public health genomics to a broader scope as the field has evolved to include diverse individual- and population-level data to help better target interventions to improve public health.” [3]

##### Dr. Bruce Korf, “Genomics in Public Health”

Dr. Bruce Korf presented the opening plenary session on genomics in public health research. Dr. Korf described how the dramatic reduction of genomic sequencing cost has opened the door to broader use of genomic medicine, and he presented examples of public health and genomic rare disease programs at his institution (The University of Alabama Undiagnosed Disease Program, the Rare Disease Discovery Ecosystem, the Alabama Genomic Health Initiative, the All of Us Research Program [1], and the BabySeq2 study) [4, 5]. Dr. Korf noted the role of genomics in offering accessible diagnoses to individuals who might not otherwise have access to experts with knowledge of specific rare diseases. He reported on the University of Alabama’s Genetics Department’s experience delivering services that are responsive to community needs and promoting equitable access to genomic medicine. He closed his presentation by looking to the future of public health genomics, considering the impact of reduced cost and improved technology, balanced with the need to demonstrate clinical utility in primary and specialty care spaces, plan for implementation and sustainability, and prioritize clinician education.

#### Speaker session 1

##### Dr. Nora Pashayan, “Implementation of Precision Breast Screening Program”

Dr. Pashayan dived more deeply into a PPH intervention and its implementation: a precision breast cancer screening program stratifying a population into risk groups and targeting prevention based on risk. Dr. Pashayan described key program considerations, including defining risk factors and risk thresholds, public health strategies, optimal outcomes, and implementation strategies. She noted examples of risk-stratified screening strategies, acceptable trade-offs, and types of evidence needed to define risk strategies and make policy decisions related to screening. People with pathogenic genetic variants in *BCRA1* or *BCRA2* are at elevated risk for breast cancer, and they may benefit from earlier and more frequent mammograms or MRI screenings. While screening can save lives by detecting breast cancer early, it also carries the risk of overdiagnosis (detecting cancers that would not have caused harm during the patient's lifetime). Acceptable trade-offs involve balancing the reduction in breast cancer mortality with the potential psychological and physical harms from treating indolent tumors. Dr. Pashayan highlighted the importance of considering implementation at all stages of research design and data collection. Policy decisions about risk-stratified strategies require epidemiological data, randomized controlled trials, modeling studies, and cost-effectiveness analyses. She noted the fine balance required to avoid “premature translation” while also ensuring innovations are not “lost in translation”. Dr. Pashayan concluded that precision screening can improve the benefit-harm balance and cost-effectiveness of screening programs, but it requires a systems approach to account for specific implementation contexts.

##### Dr. John Mathers, “Diet, Lifestyle, Behavioral Risk Factors for Precision Public Health”

Dr. John Mathers presented on the importance of PPH approaches in nutrition. Diet has documented effects on mortality. Diet’s role in mortality is likely underestimated, in part due to self-reported measurement error and lack of data on outcomes for other relevant conditions impacting diet, such as dementia. Dr. Mathers described two studies (Food4Me [2] and MedEX-UK [3]) that tested the feasibility of precision approaches to improving nutrition and other lifestyle factors. The Food4Me study demonstrated that a personalized, internet-based approach to nutrition was effective at improving dietary intake and markers of health, including body weight and waist circumference. However, using complex types of information such as phenotypic or genetic data to inform the nutritional personalization did not increase the benefits. The MedEX-UK study was a feasibility study of an interactive, web-based intervention to improve diet and physical activity outcomes among adults aged 55–74 years at increased risk of dementia. The intervention led to improved eating behaviors and cognitive function. There was no significant effect on physical activity. Dr. Mathers discussed limitations, such as the study running during COVID–19 pandemic lockdowns, which limited participants’ ability to engage in physical activity in public spaces. Finally, Dr. Mathers discussed a systems approach to precision cancer prevention interventions. He highlighted the importance of considering factors beyond genetics, such as lifestyle and diet, when offering individualized approaches in future interventions.

##### Dr. Micheal Kosorok, “The Precision Health Artificial Intelligence Research (PHAIR) Lab”

Dr. Micheal Kosorok presented on the Precision Health Artificial Intelligence Research (PHAIR) lab at the University of North Carolina at Chapel Hill. Dr. Kosorok and his team develop artificial intelligence (AI) and machine learning (ML) tools for precision health applications. The lab consists of around thirty members, ranging from high school students to post-doctoral fellows and faculty with diverse backgrounds in biostatistics, epidemiology, nutrition, statistics, public health, and others. Their projects use a range of data types including genetics, imaging, sociodemographic information, and clinical history to inform biomarker discovery, disease prediction, and dynamic treatment regimes. Dr. Kosorok described using AI to find the optional algorithm for assigning cancer treatment and using ML to understand health disparities in diabetes outcomes. He also presented on developing an algorithm for assigning treatments to individuals with knee osteoarthritis that resulted in a significant increase in positive treatment outcomes [4]. To close his presentation, Dr. Kosorok briefly summarized other ongoing projects, including Biomarkers for Evaluating Spine Treatments (BEST), and predicting gestational age based on ultrasound images [5].

#### Speaker session 2

##### Dr. Sarah Knerr, “Using Implementation Science to Achieve Population Benefit in Hereditary Cancer Screening”

The driving question behind Dr. Sarah Knerr’s work is, “how can we ensure that the excitement, energy, [and] money that is going into genomics is really leading to population-level health benefit?” Dr. Knerr shared conclusions from qualitative interviews with healthcare system staff in the United States regarding their organizations’ implementation of clinical guidelines for Hereditary Breast and Ovarian Cancer or Lynch Syndrome testing. Health systems are building one-off tools for population-based screening rather than developing and disseminating shared implementation tools. Managing organizational turnover, enabling trusting relationships among operational leaders and administrators across systems, and improving clinical guideline buy-in from staff and clinicians are essential for implementation success. Dr. Knerr noted the need to specify guidelines for population-based hereditary cancer screening, develop consensus on how screening should be done, study implementation through pragmatic trials, and address implementation barriers. Finally, Dr. Knerr noted the importance of defining additional structures to support clinical champions working to integrate hereditary cancer screening into practice.

##### Dr. Deborah Cragun, “The Value of Coincidence Analysis (CNA) and Implementation Science in Precision Public Health”

Dr. Cragun illustrated how Coincidence Analysis (CNA) can reveal how complex contextual factors may impact implementation outcomes using relatively small- to medium-sized participant samples. While prophylactic mastectomy is recommended for women with highly penetrant pathogenic variants (such as in *BRCA1* or *BRCA2*), over half of individuals with a moderate risk variant had bilateral mastectomies, in most cases exceeding care recommendations in clinical guidelines. Based on patient interviews, reasons to exceed guideline-directed care included lower trust in medical providers’ recommendations, desire to prevent cancer, and high anxiety and high fear related to cancer. The study team used CNA to understand how these factors interact to drive treatment decisions. For women with high fear and high anxiety, high trust in medical providers can protect them from pursuing unnecessary surgeries. Dr. Cragun presented a second example illustrating the role CNA can play in understanding multi-level factors that relate to the implementation of routine Lynch Syndrome screening. CNA is a valuable method to understand interacting factors that contribute to implementation of evidence-based programs as well as de-implementation of low-value services.

##### Dr. Stephanie Best, “Implementation Science in Precision Public Health”

Dr. Best described key implementation concepts relevant to public health programs: context, co-design, implementation, evaluation, sustainability, and collaboration. She described how these concepts have informed her team’s contributions to Mackenzie’s Mission [6], an initiative to inform and support the implementation of population-level adaptive reproductive genetic carrier screening across Australia. She described her team’s research on the patient recruitment pathway and strategies to collect and integrate key insights from healthcare professionals, particularly to inform strategies that support these key stakeholders in the implementation and sustainability of interventions. To develop and implement sustainable and effective interventions, Dr. Best noted the importance of involving partners in co-design, planning evaluation early, collecting a range of data, treating implementation science as a team sport, and preparing for unanticipated outcomes.

#### Closing plenary speaker

##### Dr. Rachel Shelton, “Opportunities and Considerations for Advancing Health Equity through Application of Implementation Science in Precision Public Health”

Dr. Rachel Shelton invited attendees to consider how implementation scientists can be more proactive and explicit in promoting equity through their work. Dr. Shelton highlighted the range and combination of social dimensions across which inequities exist in genomic medicine and PPH, such as sexual orientation, socioeconomic status, poverty, age, and geography. Once programs reach the implementation stage, researchers and practitioners may assume that there is evidence for effectiveness across various populations in need. However, evidence of intervention appropriateness across populations often does not exist. Health equity has not been centered in implementation science. For a 2011 review [7], Dr. Shelton and her research team were unable to find literature describing issues of power or structural racism in the field of implementation science. To apply implementation science with a health equity focus, scientists must consider equity and engagement at all stages of the translational research continuum, extending an anti-racism lens to the implementation of PPH interventions [7]. She summarized her team’s conceptual model and its relevance for other researchers. The model includes reflecting on potential bias or gaps in evidence, engaging with diverse partners early and often to co-design research questions and interventions, and considering cultural adaptations to address the needs of specific populations or social determinants of health. Finally, Dr. Shelton presented the group with theories, models, and frameworks to guide implementation determinants and outcomes with an equity focus.

### Workshop overview and structure

The virtual workshop was designed to address key challenges in PPH and foster collaboration among a diverse range of participants. The two main activities were focused on (1) identifying key outcomes and stakeholders to evaluate PPH interventions and (2) engaging underrepresented populations in PPH research and practice. These sessions collectively aimed to develop new partnerships, generate data for ongoing projects, and explore opportunities for advancing anti-racism research in PPH.

#### Activity 1: Identifying key outcomes and informant groups

The goal of this activity was to generate a list of key outcomes for evaluating PPH interventions and to discuss which types of partners should be involved in establishing a framework for evaluating PPH interventions.

Participants were asked to discuss what various decision-makers would want to know when considering potential program expansion or implementation in new settings, and what types of outcomes may be relevant across intervention types, data inputs, and contexts. Outcome themes included:

- *Clinical and Health*: Mortality rates, intermediate clinical outcomes, personal utility, and psychosocial outcomes were recognized as key indicators to assess the effectiveness of PPH.
- *Equity and Access*: Equity was a central focus, particularly program reach for underrepresented populations. Participants highlighted the need for broader research inclusion and understanding barriers to equitable program reach and uptake.
- *Implementation and Feasibility*: Workshop participants also emphasized the importance of collecting data on the feasibility and acceptability of PPH interventions across various levels (e.g., providers, policymakers, patients). The group discussed practical aspects such as implementation feasibility for providers and policymakers, as well as the acceptability and uptake of PPH interventions by diverse stakeholders.
- *Unintended Consequences*: Participants noted the need to monitor potential unintended negative consequences of PPH, such as privacy breaches, stigma, discrimination, adverse events, and spillover effects in clinical settings.

Participants recognized the importance of capturing diverse perspectives in establishing a framework for outcomes reporting from PPH programs. Special attention was given to identifying groups with expertise on reasons for not participating in PPH interventions, the patient experience, and how community leaders, patient advocates, and healthcare administrators can contribute to the successful implementation of PPH interventions. While relevant partners will depend on the context, participants listed the following potential partners:

- *Healthcare system*: primary care and genetics clinicians, healthcare administrators and leaders, patient navigators, middle management public health workers/healthcare workers
- *Community partners*: community health workers, community leaders (religious/geographic), community organizations, school-based leaders, champions of PPH
- *Researcher enterprise*: data managers/informatics/registries, health economists, research staff, research funders, industry members
- *Patient representatives*: patient advocates, patients and family members
- *Policy makers*: governmental policy makers, healthcare payers, public health directors

#### Activity 2: Engaging underrepresented populations in PPH

Activity 2 focused on addressing the challenge of engaging historically marginalized and underrepresented populations in PPH research and practice. Participants discussed strategies to ensure that these populations are not only included but play an active role in shaping the research agenda. The goals of this activity were to identify ways to disseminate findings from existing work and identify new potential work streams for health equity-focused PPH efforts [6, 7].

Several key outcomes were noted:

- *Community Engagement*: There was consensus on the need to actively involve non-researchers, including community leaders, patient advocates, and public health workers, in planning PPH research. Participants emphasized the importance of early and sustained engagement to build trust and ensure the relevance of research to underserved communities.
- *Co-design and Adaptation of PPH for Underserved Populations*: Participants considered different approaches such as deliberative democracy and engaging community partners in early stages of decision-making processes to better adapt and implement interventions based on unique population needs.
- *Building Capacity and Awareness*: To foster long-term engagement and capacity building, participants discussed establishing a grant-writing group to explore funding opportunities for projects focused on equity in PPH. There were also recommendations for expanding future conferences to include a broader range of voices, such as community health workers, students, and global representatives.
- *Dissemination strategies*: A variety of educational and dissemination strategies were proposed, including a webinar series on health equity, a future pre-conference workshop aimed at training participants on equity issues in PPH, and pursing new partnerships. Additionally, participants recommended using platforms such as social media, podcasts, and news articles to reach broader audiences and share workshop findings

### Evaluation

Half of respondents learned of the conference from colleagues or word of mouth; about a quarter learned of it from email and another quarter from social media. Respondent attendance at presentations ranged from 36–69%, indicating that attendees were coming and going throughout the day. 29% of respondents attended the virtual networking lunch during the virtual conference. 64% of respondents attended the virtual workshop.

Respondents rated the presentations highly, with almost universal agreement or strong agreement that the information provided was of high quality, presenters had very good or excellent knowledge of the subject, the sessions advanced understanding of PPH, and the sessions covered material that would be useful to respondents’ work. Workshop participants rated the information provided, facilitator knowledge, and facilitator guidance of discussions as very good or excellent. Respondents agreed or strongly agreed that the workshop helped develop PPH research priorities and that the content was relevant to their own work.

In overall assessments, all categories were rated as good (3), very good (4) or excellent (5) on a scale from one to five. Respondents rated the quality of the virtual facilities, speakers, and overall meeting experience as very good or excellent (mean 4.7, 4.6, and 4.5 out of 5). Respondents rated the workshop as good, very good, or excellent (mean 4.4 out of 5). The virtual poster session was rated 4.25 out of 5. The availability of networking was rated slightly lower, distributed from good, very good, or excellent (mean 4.1 out of 5). Respondents all agreed or strongly agreed that “the conference met its stated objective to address research, training, and networking opportunities for early career faculty and trainees in precision public health” (4.6 out of 5). Respondents all agreed or strongly agreed that they were satisfied with their overall conference participation (4.6 out of 5) and that their attendance would make them more likely to engage with PPH (4.5 out of 5) (Table 2).

**Table 2:** Evaluation ratings

**Evaluation criteria**	**Mean**	**Standard deviation**
**Speaker Sessions (*n*** **= 22)**		
Quality of Information Provided	4.55	0.54
Quality of Visual Aids	4.55	0.58
Presenter’s Knowledge of the Subject	4.85	0.36
Presenter’s Presentation Skills	4.64	0.53
Quality of Overall Presentation	4.64	0.49
This Session Advanced My Understanding*	4.64	0.49
This Session Covered Relevant Material*	4.49	0.69
**Consensus Building Workshop (*n*** **= 9)**		
Quality of Information Presented	4.44	0.53
Quality of Visual Aids	4.44	0.53
Facilitators’ Knowledge of the Subject	4.67	0.50
Facilitators’ Ability	4.56	0.53
Quality of Overall Consensus Workshop	4.33	0.71
Research Priorities	4.56	0.53
Consensus Was Useful*	4.44	0.53
**Overall Meeting Experience (*n* = 12)**		
Overall Quality of Virtual Facilitation	4.73	0.46
Overall Quality of Speakers	4.67	0.49
Overall Quality of Consensus Building	4.53	0.83
Overall Quality of Poster Session	4.33	0.98
Overall Availability of Networking	4.00	0.76
Overall Meeting Experience	4.6	0.63
I Feel That the Conference Met Its Standards*	4.6	0.51

Likert Scale: 1 = Poor, 2 = Fair, 3 = Good, 4 = Very Good, 5 = Excellent, *Likert Scale: 1 = Strongly Disagree, 2 =Disagree, 3 = Neither agree or disagree, 4 = Agree, 5 = Strongly Agree

In open-ended feedback, respondents stated that the presentations were timely and provided useful insights, especially on how to improve PPH implementation. Respondents appreciated the combination of presentations and discussion, and they recommended additional panels or discussion among speakers. Challenges included managing time zones that resulted in early or late presentations for some attendees. One respondent recommended extending the conference over more than one day to allow for additional discussion and interaction.

## Discussion

The 2023 Transdisciplinary Conference for Future Leaders in Precision Public Health “*Applying Implementation Science to Precision Public Health*” successfully convened experts and leveraged the PPHN member expertise and connections. Through systematic planning, effective communication, and targeted follow-up, the event facilitated collaborative relationships and disseminated knowledge to advance public health. Attendees were actively engaged with speakers and each other through chat and Q&A opportunities, and the conference presentations are now openly accessible online.

Several key themes emerged from the conference. Implementation science provides useful theories, models, and frameworks to guide the design, evaluation, and modification of PPH programs. Applying implementation science strategies with an equity focus will help to ensure that interventions reach underserved populations and lead to meaningful public health improvements. For example, genomic population screening and diagnostic testing integrated into primary care and public health settings can expand access to rare disease diagnosis beyond specialty clinics. Risk stratification can target interventions and screening to subpopulations most likely to benefit, aiding in early diagnosis while reducing screening-related harms and unnecessary interventions. Some personalized interventions, such as tailored dietary coaching, can be more effective than generalized strategies, without requiring significant additional resource investment. Implementation science can also play a vital role in the de-implementation of low-value or ineffective interventions. Public engagement was another resounding theme, with an emphasis on co-designing programs with community members, patients, healthcare providers, and other partners to ensure effective and sustainable implementation.

The workshop provided opportunities for participants to generate ideas and offer input to directly influence future PPHN activities. This input has helped to shape future meeting agendas, interim activities, and plans for ongoing research projects. Structured breakout sessions and open discussions allowed attendees to network and share perspectives, supporting PPHN’s mission of building a collaborative research community.

The PPHN also took steps to broaden the scope of PPH beyond its traditional focus on genomics. While genomic data remains an essential tool for stratifying populations and tailoring interventions, the conference emphasized the potential of diverse big data sources to inform PPH strategies, such as metabolic responses, geospatial data, and environmental exposures. Expanding beyond genomics-focused research is a priority of the PPHN, and future activities will continue to prioritize engaging researchers from a range of PPH fields.

The 2023 conference and workshop advanced the PPHN’s goals of building community, identifying areas of work, and exploration of implementation science approaches in the field. By providing a forum for early- and mid-career researchers to explore PPH challenges and build interdisciplinary networks, the event strengthened the foundation for ongoing collaboration. Future conferences will build on this momentum, creating new opportunities to advance the field and expand the reach and impact of PPH programs.

## Data Availability

Data are available upon request to the corresponding author.
